# Use of fish species from different trophic levels to control algae and water quality: An enclosure experiment in eutrophic area of Xiaojiang River

**DOI:** 10.1371/journal.pone.0171953

**Published:** 2017-03-08

**Authors:** Lian Hu, Zhi Yang, Xiaojie Pan, Na Zhao, Jianhua Peng, Chengyan Wan

**Affiliations:** 1 Key Laboratory of Ministry of Water Resources for Ecological Impacts of Hydraulic-projects and Restoration of Aquatic Ecosystem, Institute of Hydroecology, Ministry of Water Resources & Chinese Academy of Sciences, Wuhan, China; 2 Hubei Provincial Collaborative Innovation Center for Water Resources Security, Wuhan, China; University of Hyogo, JAPAN

## Abstract

The effects of stocking both filter-feeding fish and piscivorous fish were compared to the effects of stocking only filter-feeding fish for suppressing algal blooms and improving water quality in the impoundment area of Xiaojiang River where catfish were dominant. Using only filter-feeding fish for algal suppression and water quality control was more effective in the short-term, but use of both filter-feeding fish and piscivorous fish was better in the long-term. Obvious suppression of phytoplankton biomass (PB) only occurred during the first 14 days regardless of the fish stocked. Adding fish to the enclosure clearly alters phytoplankton community structure and introducing piscivorous fish to an enclosure stocked with filter-feeding fish changed the relative densities of dominant algae species. While stocking filter-feeding fish decreased total nitrogen concentration by removing phytoplankton, it did not effectively decrease total phosphorus and Chlorophyll a concentrations. Introducing piscivorous fish to the enclosure weakened the relationship between nutrients and phytoplankton. Results indicate that stocking only filter-feeding fish to improve water quality and suppress phytoplankton in an impoundment area is insufficient and other technologies and means should be applied simultaneously.

## Introduction

Dam construction significantly modifies river ecosystems [1–2]. The resulting impoundment affects the physical, chemical, and biological characteristics of a river and is a primary contributor to the algal blooms and water quality deterioration that often occurs after damming [3–5]. The impoundment-induced changes in hydrodynamics will produce a eutrophication problem if there is excessive nutrient loading to the impounded water [4, 6], followed by algal blooms [7–9]. The close relationship of eutrophication with water quality degradation and algal blooms has been widely recognized [8, 10–12]. Excessive nutrient loads lead to harmful algal blooms (HABs), which degrade water quality[13,14] and can produce toxins that affect fish and other taxa survivals [15–17].

Biomanipulation methods are frequently applied to control algal blooms by reducing algal biomass[18–20] and the use of filter-feeding fish (e.g. silver carp, Hypophthalmichthys molitrix) and mollusks (e.g. *Dreissena rostriformis bugensis*) to produce a top-down effect on phytoplankton communities has been well-documented[19,21,22]. In recent years, the simultaneous use of phytoplanktivorous and piscivorous fish to reduce algal biomass has been used more frequently in biomanipulation experiments [20, 23]. The cascading effects of fish predation and zooplankton grazing overcomes the food-preference limitation imposed by using only planktivorous fish[24–25]. Radke and Kahl [24] concluded that silver carp should be used for biomanipulation only if the primary aim is to reduce nuisance blooms of large phytoplankton species and Wang et al. [25] reported that silver carp are not recommended for phytoplankton control when the objective was to control the entire phytoplankton community of a shallow lake. However, biomanipulation experiments carried out in a reservoir using simultaneous release of filter-feeding and piscivorous fish are rare, especially studies that compare the effectiveness of stocking fish from both trophic levels with the use of filter-feeders only.

In the past decade, a large number of hydropower stations with capacities ranging from several MW to over 20,000 MW have been constructed in China. Among the new hydropower stations, the Three Gorges Project (TGP) has been well-studied because of numerous contentious environmental issues [26]. With the completion of the TGP in 2012, the Three Gorges Reservoir (TGR) stretched from Sandouping in Hubei Province to the Jiangjin District of Chongqing (~660 km) and backed into over 100 tributaries. Since the TGR filled to 135 m in 2003, environmental consequences have emerged [26] and local managers began facing algal blooms and deteriorating water quality in many tributary bays[27]. A nearly continuous algal bloom emerged on the impounded section of Xijiang River, a primary tributary of the TGR[27].

With a drainage area of 5173 km^2^ and mainstem length of 182 km, the Xiaojiang River is the largest primary tributary in the middle north shore of TGR. Since the water level of TGR rose to 172 m in 2008, the backwater area of Xiaojiang River extends to Hanfeng lake of Kaixian County town (nearly 90 km in length), of which about 40 km is permanently inundated (also called Xiaojiang Bay) as the TGR water level fluctuates from 145 m to 175 m. Xiaojiang River has the largest submerged area and the most extensive riparian area in the TGR system [28]. The backwater area of Xiaojiang River has also developed the highest algal biomass[29], high-frequency algal blooms and is dominated by piscivorous fish species (*Silurus asotus*, *Culter ilishaeformis*, *Culter mongolicus*)[27]. For these reasons, the Xiaojiang River backwater is an appropriate location to carry out a biomanipulation experiment designed to control algae and improve water quality.

Two species of filter-feeding fish, silver carp *Hypophthalmichthys molitrix* and bighead carp *Aristichthys nobilis*, and one species of piscivorous fish, the catfish *Silurus asotus*, were selected for the enclosure experiment. The effectiveness of using all three fish species was compared to that of using only the two filter feeders for algal control. The feasibility of using biomanipulation to suppress algae and improve water quality was then assessed for Xiaojiang Bay, an impoundment in which the dominant piscivore is the catfish [28].

## Methods

All experimental procedures were conducted in conformity with institutional guidelines for the care and use of laboratory animals, and protocols were approved by the Institutional Animal Care and Use Committee in Institute of Hydroecology, Ministry of Water Resources & Chinese Academy of Sciences, China.

### Experimental site

**Fig 1:**
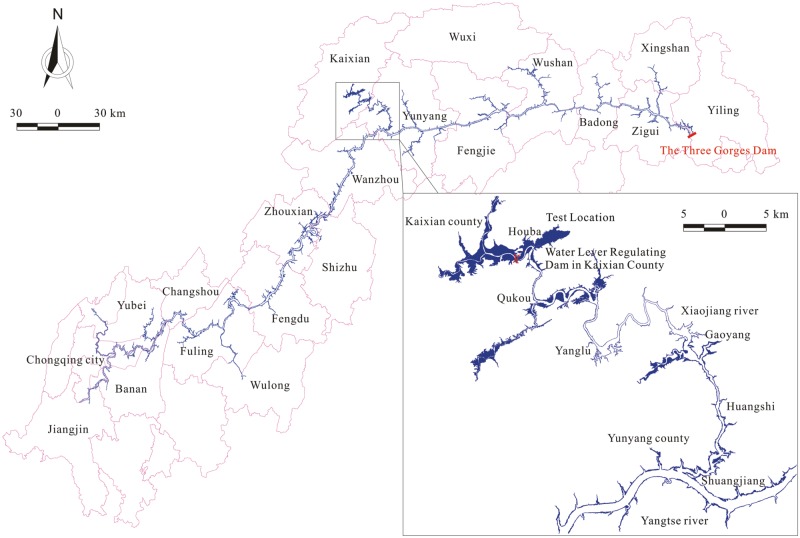
Location of the experimental site.

The experimental site was located between the towns of Houba and Qukou (Fig 1), the section of the Xiaojiang backwater with the highest chlorophyll a value and the highest Cladocera density (160 ind/L) and biomass (60 mg/L) [29]. The experimental site was eutrophic, with total nitrogen (TN) and phosphorus (TP) ranges of 1.44–3.26 mg/L and 0.10–0.12 mg/L [30]. On both shores of the experimental area there were numerous plant species, with Xanthium sibiricum, Bidens pilosa, Eclipta prostrata dominating in the water level fluctuation zone [31]. Their decomposition at high water level releases large quantities of nutrients into the water column. The backwater in the experimental area had a flow velocity of <0.05 m/s, a mean width of ~100 m and a mean depth of ~2.1 m. The experimental site had more than 50 fish species, including silver carp and bighead carp and the dominant piscivorous species, the Amur catfish [28]. There were no specific permission were required for carrying out experiments in this site and also this field study did not involve endangered or protected species.

### Experimental design

We conducted our experiments in twenty-two enclosures (L×W×H = 3 m ×2 m ×2 m), which constructed of polyvinyl chloride (PVC) sheeting (6.0 cm thickness) fastened to frames made of steel tubing (5.0 cm). The enclosures were placed 25 m off the north shore at a water depth of 1.5 m and the experimental period was June 11-July 23, 2013. The tops and bottoms of the enclosures were open to maintain light intensity and free exchange of nutrients [32]. The lower end of the enclosure frames were fixed with rocks and buried to a depth of 20 cm in the sediment and the frame tops extended 30 cm above the water surface. Prior to treatment, the PVC sheeting was pulled down near, but not touching, the sediment for 5 days [32] to allow the water quality and plankton community to stabilize. After stabilization, the PVC was fastened to the steel tube frame, creating an enclosure volume of ~13.5 m^3^.

Ten treatments and a control group [Control] were prepared. The first five treatments were combinations of silver carp and bighead carp [T1-T5] and the second five treatments were combinations of these two ones and the catfish [T6-T10]. The treatments were arranged as shown in Table 1, based on Xie [33]and preliminary studies conducted on the Xiaojiang River. Each treatment was carried out in duplicate (two enclosures/treatment). The mean weight of fish released into enclosures was ~3 g for silver carp and bighead carp and ~8 g for catfish.

**Table 1:** Experimental designs for different treatments.

Treatment	Stocking weight each fish species (g)			Stocking density(g/m^3^)
Control	Silver carp	Bighead carp	Catfish	
**T1**	81	189	0	20
**T2**	121	283	0	30
**T3**	162	378	0	40
**T4**	202	472	0	50
**T5**	243	567	0	60
**T6**	81	189	27	22
**T7**	121	283	40	33
**T8**	162	378	54	44
**T9**	202	472	68	55
**T10**	243	567	81	66

### Physical, chemical and biological parameters of water in the enclosures

The first sampling for physical, chemical and biological parameters was carried out on June 11, immediately before the fish were introduced to the enclosures, and the enclosure water was resampled every seven days, giving a total of seven sample sets collected one week apart. Before collecting samples, water temperature (WT, ℃), dissolved oxygen (DO, mg/L) and pH were measured *in situ* using a multifunctional YSI meter (YSI-650, YSI Inc., Yellow Springs, OH). Samples for total nitrogen (TN, mg/L), total phosphorus (TP, mg/L) and Chlorophyll a (Chl-a, mg/L) were collected from each enclosure by the method described by Zhu *et al* [32]. Water samples were immediately placed on ice and taken to the laboratory for analysis using standard methods (Environmental Quality Standards for Surface Water, GB3838-2002). One liter of water was sampled using a 2 L organic glass hydrophore, preserved by adding 15 mL Lugol’s solution and transported to the laboratory for analysis of phytoplankton biomass (PB) using the method described by Gibson *et al*. [34], herein the cell number of different phytoplankton species were directly counted in a plankton counting chamber (0.1 mL) using an optical digital microscope with different magnifications and meanwhile[35] the phytoplankton species were identified referring to the book Freshwater Algae in China [36].

### Data analysis

The values of the environmental parameters for each treatment for each set of samples were obtained by averaging values from the duplicate enclosures. During the experimental period from the second sampling to the last sampling, the mean values and standard deviations of environmental parameters for each treatment were calculated and then the mean values between each fish treatment and control treatment were compared by using the method of Independent Samples T Test [37]. Two-way analysis of variance (Two-way ANOVA) was used to examine the influences of different treatment types(no fish, stocking sliver carp and bighead carp, and stocking sliver carp, bighead carp and catfish) and different sampling times on the variations of water quality and phytoplankton biomass[37]. The variation values (Varv_*i*th_) of water quality and the phytoplankton biomass at the second sampling to the seventh sampling for a given treatment were respectively calculated as follows:

$${\text{Varv}}_{i}{}_{\text{th}}={\text{V}}_{i}{}_{\text{th}}-\text{Firv}$$

Where V_*i*th_ was the value of an environmental parameter at the *i*th sampling, Firv was the value of an environmental parameter at the first sampling.

Based on the method of Hedges *et al*. [38], the presence/absence (P/A) value for each parameter at each sampling time was obtained by taking the common logarithm of the ratio of the value for each treatment (X_present_) and the corresponding value for the control group (X_absent_), as expressed below:

$$\text{P}/\text{A }=\text{ log}10\;\left({\text{X}}_{\text{present}}/{\text{X}}_{\text{absent}}\right)$$

Where X_present_ and X_absent_ were the values of environmental parameters when fish were present and absent [38]. If the P/A value of a parameter for a treatment is < 0, the parameter value with the treatment was less than the corresponding control. Because the initial P/A values (first sample) were different for each parameter, the initial P/A values for each parameter were set to zero to allow better comparison of the trends resulting from different treatments.

Pearson’s correlation analysis was performed on pairs of water quality parameters[37]. All the statistical analyses were performed using SPSS (Ver.16.0, SPSS Inc., USA). Figures were made using CorelDRAW 12 and Excel 2010.

## Results

### Effect of treatments on physical, chemical and biological parameters of water

During the experimental period from the second sampling to the last sampling(W1-W6), water temperature varied from 21.4°C to 29.6°C, with a mean value of 24.2°C. After the first sampling, the mean value of each water quality parameter was shown in Table 2. The mean concentration of DO, pH, TN, Chl-a and PB in fish treatments were not significantly different from the control group (Table 2), but the differences between T2-T10 and the control group in TP were significant.

**Table 2:** Mean values of environmental parameters for each treatment (Mean±std.).

Treatment	DO(mg/L)	pH	TN(mg/L)	TP(mg/L)	Chl-a(μg/L)	PB(mg/L)
**Control**	6.79±3.18	8.09±0.18	0.95±0.26	0.07±0.01	41.94±13.12	2.65±2.77
**T1**	7.09±3.62	8.30±0.35	0.69±0.27	0.06±0.02	33.75±11.92	2.35±0.91
**T2**	5.98±3.21	8.27±0.38	0.74±0.24	0.05±0.02*	37.28±9.94	2.61±0.89
**T3**	6.95±3.60	8.29±0.38	0.70±0.29	0.05±0.01*	34.58±14.39	1.54±0.61
**T4**	6.06±3.30	8.27±0.34	0.82±0.30	0.06±0.02	34.55±9.21	2.12±0.70
**T5**	6.45±3.69	8.27±0.05	0.80±0.32	0.06±0.02	34.43±11.01	2.38±0.90
**T6**	6.08±3.47	8.21±0.28	0.85±0.25	0.06±0.01*	36.45±9.17	2.90±0.84
**T7**	6.09±3.56	8.27±0.30	0.91±0.22	0.06±0.01*	36.06±8.89	2.05±1.30
**T8**	5.67±3.27	8.28±0.32	0.87±0.26	0.05±0.02*	31.89±9.23	2.03±1.93
**T9**	6.76±3.47	8.29±0.34	0.71±0.20	0.05±0.01*	31.22±7.73	2.16±1.19
**T10**	6.53±3.03	8.29±0.33	0.80±0.22	0.05±0.01*	31.55±6.05	2.12±0.80

Notes:

* indicated significant differences between the treatment and the control (*p*<0.05) and were in bold print

Results from the Two-way ANOVA were shown in Table 3. The differences of sampling times had the significant effects on the variations of DO, pH, TN, TP, Chl-a and PB concentrations (*p*<0.05), while the differences of treatment types led to significant statistical differences on the variations of pH, TN, TP and Chl-a concentrations(Table 3). However, the differences of treatment types didn’t cause the obviously differences on the variations of DO and PB concentrations (*p*>0.05, Table 3). Additionally, the interactions between treatment type and sampling time on the variations of all environmental parameters were significant (*p*<0.05, Table 3).

**Table 3:** Results of Two-way analysis of variance (Two-way ANOVA) examining the effects of different treatment types and the different sampling times on the variations of environmental parameters.

Variable	Factor	*F*	*p*
DO(mg/L)	Treatment type	2.517	0.091
	Sampling time	153.968	**0.000**
	Treatment type and Sampling time	3.408	**0.002**
pH	Treatment type	19.301	**0.000**
	Sampling time	83.890	**0.000**
	Treatment type and Sampling time	4.028	**0.000**
TN(mg/L)	Treatment type	7.278	**0.002**
	Sampling time	19.434	**0.000**
	Treatment type and Sampling time	2.523	**0.016**
TP(mg/L)	Treatment type	8.910	**0.001**
	Sampling time	6.494	**0.000**
	Treatment type and Sampling time	3.671	**0.001**
Chl-a(μg/L)	Treatment type	8.326	**0.001**
	Sampling time	20.707	**0.000**
	Treatment type and Sampling time	7.383	**0.000**
PB(mg/L)	Treatment type	1.108	0.338
	Sampling time	29.904	**0.000**
	Treatment type and Sampling time	4.868	**0.000**

Note:*p* < 0.05 was considered significant and highlighted in bold

### P/A values for water quality parameters

**Fig 2:**
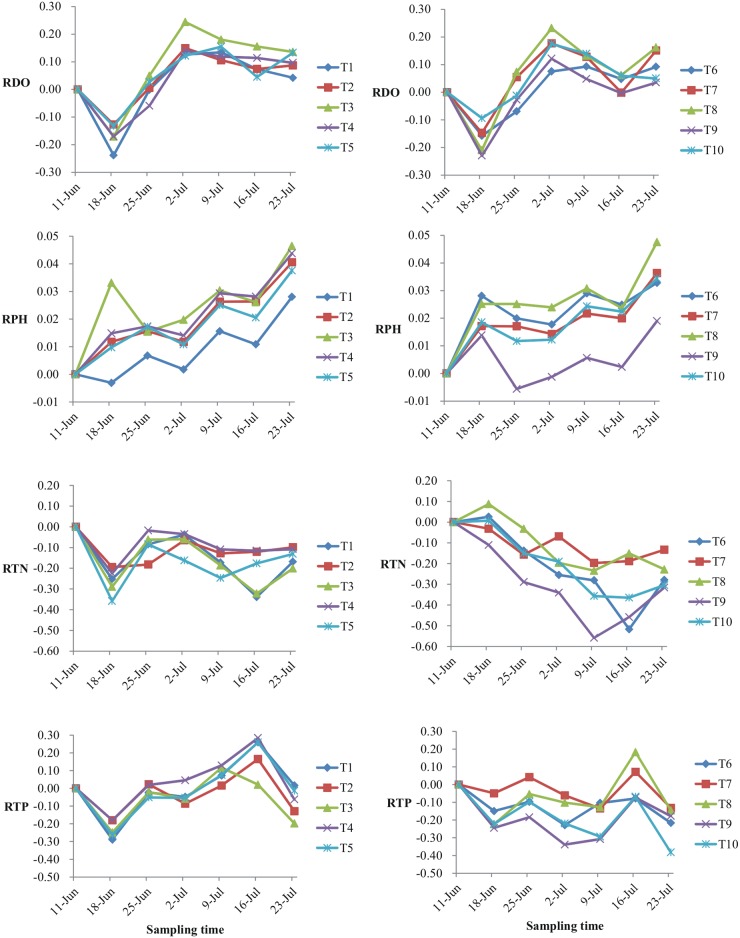
P/A values for DO (RDO), pH (RPH), TN (RTN) and TP (RTP) for each treatment.

The P/A values for DO, pH, TN and TP for all treatments were shown in Fig 2. In all ten treatments, the P/A values of DO (RDO) decreased for the first week (before June 18), increased rapidly to the maximum value (0.07–0.24) in the subsequent two or three weeks and then fluctuated, ranging from 0.03 to 0.16 by the end of the experiment. The P/A values of pH (RPH) fluctuated but displayed an upward trend and the pH for all treatments increased during the last week of the experiment. The P/A values for TN (RTN) for treatments T1-T5 fluctuated after initially decreasing, while the RTN for treatments T6-T10 displayed a more consistent downward trend. The P/A values of TP (RTP) for all 10 treatments decreased during the first week, with larger decreases for T1-T5. All treatments, except for T3, reached maximum values at week 5 (July 16) and TP in all treatments decreased over the last week of the experiment. RTP for T6-T10 also trended downward more than for T1-T6. Moreover, the RTPs of T6, T9 and T10 were all <0 during the entire experimental period, while for treatments T1-T5, the RTPs were all >0 at week 5 (July 16).

### P/A values for Chlorophyll-a and phytoplankton biomass

**Fig 3:**
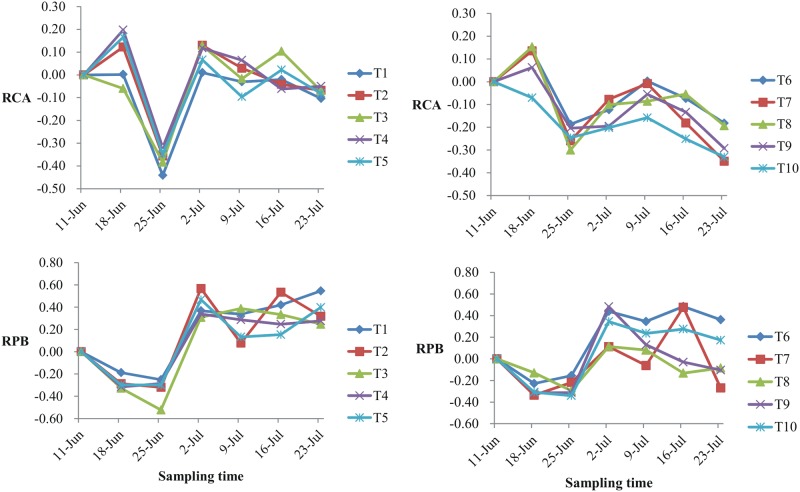
P/A values for Chlorophyll a (RCA) phytoplankton biomass (RPB) for all of fish presence treatments.

**Fig 4:**
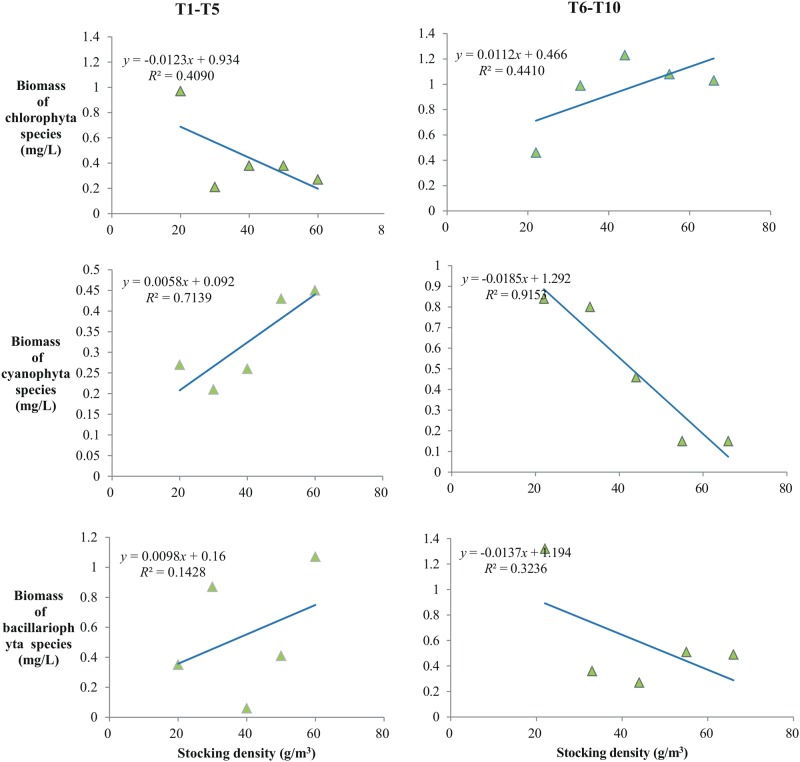
Linear correlation of stocked fish density with biomass of three dominant phyla at day 14.

The P/A values for Chl-a (RCA) and phytoplankton biomass (RPB) for all treatments are shown in Fig 3. The RCA of all treatments decreased sharply during the second week (July 25) and declined again during the last week. During week 3, the RCA for T1-T5 recovered to ≥0, while the RCA for treatments T6-T10 all remained <0. It is clear from Fig 4 that T6-T10 led to a lower final Chl-a than T1-T5.

The RPB, for all treatments, was <0 for the first 2 weeks and then increased rapidly during week 3. After week 3 the RPB fluctuated, but more noticeably for T6-T10 than for T1-T5. At the last sampling, the RPBs for T1-T5 were >0 but there were three treatments (T7, T8, T9) for which the RPBs were <0.

### Variations of phytoplankton compositions and biomasses under different stocking ways

The phytoplankton compositions and biomasses for each treatment at day 0 (initial), day 14 and day 42 (final) are shown in Table 4. During the three samplings, phytoplankton from 6 phyla was observed in the control. After the introduction of fish, the number of phyla observed on day 42 decreased in 7 of the 10 treatments and no phytoplankton from Pyrrophyta and Euglenophyta were collected. As the experiment progressed, the biomasses of different phytoplankton species varied, both in the presence and absence of fish. However, changes in phytoplankton community structure displayed very different patterns in the different treatment groups. Initially, the phytoplankton community was dominated by species of Chlorophyta, Cyanophyta, Bacillariophyta, Pyrrophyta and Euglenophyta. At day 14, the dominant species in the control and T1-T5 were species of Chlorophyta, Bacillariophyta and Euglenophyta, while in T6-T10 the dominant species were Chlorophyta, Cyanophyta and Bacillariophyta. At the end of the experiment (day 42), phytoplankton in the control group and all treatment groups included species of Chlorophyta and Cyanophyta. In addition to species from these two phyla, species of Cryptophyta and Euglenophyta were observed in the control, Cryptophyta and Bacillariophyta in T1-T5, and only Bacillariophyta in T6-T10.

**Table 4:** Phytoplankton composition and biomass (mg/L) for each treatment at day 0, 14 and 42.

	Species	Control	T1	T2	T3	T4	T5	T6	T7	T8	T9	T10
	Cryptophyta	0.19	0.12	0.07	0.00	0.24	0.06	0.18	0.12	0.06	0.01	0.00
	Chlorophyta	0.90	0.79	0.55	1.20	1.06	0.79	1.47	0.40	1.09	0.96	0.51
**Day 0**	Cyanophyta	0.78	0.74	1.01	0.38	0.87	0.82	0.61	0.49	0.30	0.89	0.56
	Bacillariophyta	1.04	0.93	2.12	0.29	0.23	1.29	0.79	0.92	0.87	0.38	0.84
	Pyrrophyta	0.71	0.61	0.00	0.31	0.92	0.92	0.92	1.53	0.92	1.83	0.92
	Euglenophyta	0.67	0.18	0.55	0.83	0.70	0.55	0.28	1.07	1.19	0.83	1.44
	Sum	***4*.*29***	***3*.*37***	***4*.*30***	***3*.*00***	***4*.*02***	***4*.*43***	***4*.*25***	***4*.*53***	***4*.*43***	***4*.*90***	***4*.*26***
	Cryptophyta	0.01	0.19	0.01	0.01	0.08	0.00	0.07	0.21	0.00	0.00	0.32
	Chlorophyta	1.71	0.97	0.21	0.38	0.38	0.27	0.46	0.99	1.23	1.08	1.03
**Day 14**	Cyanophyta	0.45	0.27	0.21	0.26	0.43	0.45	0.84	0.80	0.46	0.15	0.15
	Bacillariophyta	1.31	0.35	0.87	0.06	0.41	1.07	1.32	0.36	0.27	0.51	0.49
	Pyrrophyta	0.00	0.00	0.32	0.00	0.31	0.31	0.42	0.35	0.00	0.31	0.00
	Euglenophyta	0.92	0.15	0.49	0.21	0.52	0.18	0.00	0.11	0.34	0.40	0.00
	Sum	***4*.*39***	***1*.*93***	***2*.*11***	***0*.*92***	***2*.*13***	***2*.*28***	***3*.*04***	***2*.*81***	***2*.*29***	***2*.*45***	***2*.*00***
	Cryptophyta	0.29	1.66	0.45	0.19	0.80	0.97	0.27	0.13	0.34	0.08	0.27
	Chlorophyta	0.15	0.46	0.51	0.22	0.21	1.05	0.49	0.19	0.18	0.47	0.73
**Day 42**	Cyanophyta	0.37	0.17	0.32	0.18	0.21	0.46	0.18	0.28	0.31	0.11	0.26
	Bacillariophyta	0.11	0.68	0.97	0.50	0.68	0.30	0.98	0.02	0.09	0.30	0.18
	Pyrrophyta	0.00	0.00	0.00	0.15	0.00	0.00	0.54	0.00	0.00	0.00	0.00
	Euglenophyta	0.15	0.00	0.00	0.08	0.00	0.00	0.00	0.00	0.00	0.00	0.15
	Sum	***1*.*07***	***2*.*97***	***2*.*25***	***1*.*32***	***1*.*90***	***2*.*78***	***2*.*45***	***0*.*62***	***0*.*92***	***0*.*96***	***1*.*59***

To indicate the control exerted on PB, stocked fish density was correlated with biomass of the three dominant phyla at day 14 (Fig 4, when displayed the most obviously suppression for PB). The correlation of Cyanophyta biomass with fish stocking density was tighter (*R*^*2*^ = 0.7139 and *R*^*2*^ = 0.9153) than with Chlorophyta and Bacillariophyta. However, for T1-T5, PB increased with stocking density and, for T6-T10, PB decreased with stocking density. In T1-T5, the biomass of Bacillariophyta and Cyanophyta increased with stocking density and, in T6-T10, the biomasses of Bacillariophyta and Cyanophyta decreased with the stocking density.

### Correlations among water quality and biotic parameters

The correlations among water quality and biotic parameters for treatments T1-T5 and T6-T10 are shown in Table 5. There were significantly correlations between DO and pH, as well as Chl-a for both T1-T5 (*r* = 0.945, *p* < 0.01 and *r* = 0.720, *p* < 0.01) and in T6-T10 (*r* = 0.906, *p* < 0.01 and *r* = 0.464, *p* < 0.05). The pH also significantly correlated with Chl-a (*r* = 0.736, *p* < 0.01) for T1-T5, TN (*r* = 0.585, *p* < 0.01) and Chl-a (*r* = 0.434, *p* < 0.05) for T6-T10. Significant correlations were observed between TN and TP for T1-T5 (*r* = 0.406, *p* < 0.05) and for T6-T10 (*r* = 0.416, *p* < 0.05), and between TN and Chl-a for T6-T10 (*r* = -0.382, *p* < 0.05). However, correlations between Chl-a and TP or PB were not significant for any treatment. The only parameters significantly correlated with PB were TN for T1-T5 (*r* = -0.586, *p* < 0.01) and DO for T6-T10 (*r* = -0.411, *p* < 0.05).

**Table 5:** The correlations among the water quality and biotic parameters for the treatments T1-T5 and T6-T10.

Treatments		DO	pH	TN	TP	Chl-a	PB
	DO		0.945**	-0.165	-0.014	0.720**	-0.033
	PH	0.945**		-0.224	0.077	0.736**	-0.023
	TN	-0.165	-0.224		0.406*	-0.269	-0.586**
T1-T5	TP	-0.014	0.077	0.406*		0.200	-0.359
	Chl-a	0.720**	0.736**	-0.269	0.200		0.121
	PB	-0.033	-0.023	-0.586**	-0.359	0.121	
	DO		0.906**	-0.435*	-0.398*	0.464*	-0.411*
	PH	0.906**		-0.585**	-0.332	0.434*	-0.208
	TN	-0.435*	-0.585**		0.416*	-0.382*	-0.175
T6-T10	TP	-0.398*	-0.332	0.416*		0.300	0.085
	Chl-a	0.464*	0.434*	-0.382*	0.300		0.277
	PB	-0.411*	-0.208	-0.175	0.085	0.277	

*Significance <0.05.

**Significance <0.01.

## Discussion

In this study, an enclosure experiment was designed to determine differences in algal suppression and water quality improvement due to differences in the trophic level of fish released into an enclosure in which the phytoplankton community is dominated by species of Chlorophyta, Bacillariophyta and Cyanophyta(Table 4). Introducing fish to the enclosures did not significantly change the mean value of any water quality parameter with the exception of TP in treatment T1,T2,T6-T10 (Table 2). As fish stocking density increased in treatments T1-T10, mean PB in T1-T5 and in T6-T10 decreased respectively from 2.35±0.91 mg/L to 2.38±0.90 mg/L and from 2.90±0.84 mg/L to 2.12±0.80 mg/L(Table 2),indicating that only stocking filter-feeding fishes even if increased their stocking density also didn’t obviously decrease the mean PB level of the entire experimental period in the impoundment area of the Xiaojiang River. The result was opposite to some other study results which showed stocking filter-feeding fish was able to obviously decline the PB[20,39–41]. However, for all fish treatments, the lowest mean PB for T1-T5 and for T6-T10 was observed in T3 and in T8 respectively which the stocking density for the sliver carp and bighead carp was 40 g/m^3^, might indicating that maintained this density for these two kinds of fish species was most appropriate for controlling the PB concentration of this study area. Our results from Fig 3 also supported above point of view.

Variations along the different sampling times for pH, TN, TP, Chl-a concentrations were strongly related to the treatment types (Table 3), indicating that differences of fish stocking schemes could exert obviously effects on the variations of above four environmental parameters in the study area. This result was consistent with the point of view that nontraditional biomanipulation had the possibility to influence some water quality parameters [39–41]. However, weakly relationship between the treatment type and variation of DO and PB concentrations also was observed in Table 3, which indicated that it was not effective for controlling PB concentration by using the silver carp, bighead carp or/and catfish in this study area. Similar result also was observed by Wang et al [25]. Actually, as pointed out by Zhang *et al*. [41] and Ha et al. [42], the silver carp and the bighead carp did not work efficiently for reducing the entire phytoplankton biomass in a specifically area because of the limitation in feeding organs, and using the piscivorous fish for controlling the algal abundance was only appropriate when they could obviously decline the grazing pressure of other fishes on zooplankton.

The P/A values, displayed in Fig 2, showed that the variation of RDO and RPH with time was similar for the two types of treatment, T1-T5 and T6-T10. However, variation patterns of RTN and RTP were quite different for T1-T5 and T6-T10. The RTN with T1-T5 fluctuated more widely than with T6-T10 and the RTP curves for T1-T5 and T6-T10 decreased initially and then undulated upward for T1-T5 but downward for T6-T10. Yi *et al*. [39] once found that, after one or two weeks, the P/A values for TN and TP decreased and then rebounded if stocked with only filter-feeding fish. Our results indicated that this similar pattern changes for the treatments of stocking only filter-feeding fishes (Fig 2). However, a different changed pattern for RTN was observed if piscivorous fishes were added to the filter-feeding fish enclosures. Although nitrogen and phosphorus concentrations the enclosures would increase due to fish internal loading [39,41] and acetylcholine metabolism[19], especially in small enclosures, but obviously above reasons could not explain the variation trends referred to the treatments of T6-T10 because RTN decreased with increasing the fish stock density (Fig 2). Skov *et al*. [43] had found out that the piscivorous fish may be had a significant structuring force for changing the fish community and in turn declined the Chl-a and TP concentrations in shallow eutrophic lakes by a 7-year study. In this study, the catfish was one fish species which feeding mainly on the small fishes and benthos [44], which probably could impact the community structure of benthos and in turn exerting the effect on phytoplankton community structure [45] resulting finally in the variations of TN and TP concentrations. Sieben and Ljunggren [46] had also figured that a meso-predator release of stickleback may dramatically change coastal food web constitution through a trophic cascade, which indicated that the predator adding may can influence the flow of nutrients.

Yi *et al*. [39] reported that Chl-a and PB in fish present treatments were generally lower than in fish absent treatments during the entire experimental period in hypereutrophic Lake Taihu that displayed markedly suppressed Chl-a and PB when stocked with silver or bighead carp. Our results showed it was more effective in the short-term to stock filter-feeding fish, but better for long-term control of Chl-a to stock both filter-feeding fish and piscivorous fish (Fig 3). Wang *et al*. [25] also reported that planktivorous fish failed to decrease Chl-a in shallow lakes due to decoupling of nutrients and chlorophyll, consistent with our findings (Table 5). Direct suppression of PB was observed before week 3 regardless of stocking scheme (Fig 3). The subsequent increase in PB was probably because phytoplankton smaller than 5 μm were poorly filtered by silver carp [47] and bighead carp [48], causing an increase in the number of small phytoplankton freed from grazers and competitors [24,39,49]. Yi *et al*. [39] also noted that filter-feeding fish typically promote small zooplankton by reducing control of phytoplankton by zooplankton.

Fukushima *et al*. [50] found that stocking silver carp causes a shift in phytoplankton species towards small individual green algae due to food selection. Ha *et al*. [42] also reported that biomanipulation using piscivore and Daphnia stocking substantially changes the phytoplankton community, consistent with our results (Table 4). Different patterns of change in phytoplankton community structure with the two stocking schemes were also observed (Table 4), indicating that the different stocking schemes act by different mechanisms. Actually, due to the niche difference, filter-feeding fish suppress PB mainly by direct feeding while piscivorous fish reduce PB indirectly by the cascade effect, as reported by Skov *et al*. [43]. The results displayed in Fig 4 provided additional evidence for this point of view. As shown in Fig 4, with the two stocking schemes, the linear relationships between stocking density and PB for the three dominant phyla had opposite signs. In contrast to other studies [39,49,50], our results indicated that Cyanobacteria biomass increased and green algae decreased as the density of filter-feeding fish increased. Because of the complexity of aquatic ecosystems, determining the cause of differences observed with the two stocking schemes will require further study.

There was no obvious correlation between TP and PB with either stocking scheme, although the correlation in T1-T5 was stronger than in T6-T10 (Table 5). The removal of phytoplankton by release of fish did not decrease TP in this impoundment area. A significant correlation between TN and PB was observed in T1-T5 and not in T6-T10, indicating that the addition of piscivorous fish weakens the relationship. Biomanipulation, using only filter-feeding fish, to decrease TN and TP was inappropriate for impoundments dominated by catfish [28] because catfish weaken the relationship between TP or TN and PB.

No significant correlations were observed between Chl-a and PB for either of the two stocking schemes (Table 5). Guo *et al*. [21] showed that Chl-a was positively correlated with dinoflagellate biomass but no significant correlations with other algae, if the concentration of Chl-a in the backwater area of Xiaojiang River was greater than 19μg/L. In this study, the concentration of Chl-a of each treatment exceeded 30 μg/L and the dinoflagellate biomass was very low compared to other algal taxa (Table 4), indicating that PB is more effective than Chl-a for predicting algal blooms.

## Conclusion

The mean values of TN, Chl-a and PB did not decrease significantly, compared to the control, with either of the two stocking schemes. However, the mean values of TP for some treatments (especially for all treatments of stocking both filter-feeding fish and piscivorous fish) were significantly lower than the control group. Stocking only filter-feeding fish to control TN, TP, Chl-a and PB was more effective in the short-term while stocking both filter-feeding fish and piscivorous fish was more effective in the longer term. Nevertheless, only short-term (< 21 days) suppression of PB occurred with either stocking scheme. The change in phytoplankton community structure over time indicated differences not only between fish present and the fish absent treatments but also between the two stocking schemes. Adding fish to the enclosure clearly changed the phytoplankton community structure. Releasing piscivorous fish to the enclosure produced the opposite density effect on dominant algae species, suggesting different mechanisms of algal biomass suppression. The results also showed that stocking filter-feeding fish decreased TN concentration by removing phytoplankton while evidence for reducing TP and Chl-a concentrations was not conclusive. Introducing piscivorous fish to the enclosure weakened the relationships between nutrients and phytoplankton. Given the weakening effect produced by piscivorous fish and the dominant position of catfish in this impoundment [28], stocking only filter-feeding fish to improve water quality and suppress phytoplankton was insufficient. Other technologies and means, such as decreasing external nutrient input [51] and removing carnivorous fish [52], should be applied simultaneously.

## Supporting information

S1 Experimental Data (XLSX)

## References

[1] Gehrke PC, Gilligan DM, Barwick M. Changes in fish communities of the Shoalhaven River 20 years after construction of Tallowa Dam, Australia. River Research & Applications. 2002; 18: 265–286.

[2] Mims MC, Olden JD. Fish assemblages respond to altered flow regimes via ecological filtering of life history strategies. Freshwater Biology. 2013; 58: 50–62.

[3] Margolis BE, Castro MS, Raesly RL. The impact of beaver impoundments on the water chemistry of two Appalachian streams. Canadian Journal of Fisheries & Aquatic Sciences. 2001; 58:2271–2283.

[4] Yang ZJ, Liu DF, Ji DB.Influence of the impounding process of the Three Gorges Reservoir up to water level 172.5 m on water eutrophication in the Xiangxi Bay. Science China Technological Sciences. 2010; 53: 1114–1125.

[5] Yong HJ, Yang JS, Park K. Changes in water quality after the construction of an estuary dam in the Geum River Estuary Dam system, Korea. Journal of Coastal Research. 2014; 30: 1278–1286.

[6] Lian J, Yao Y, Ma C, Guo Q. Reservoir operation rules for controlling algal blooms in a tributary to the impoundment of Three Gorges Dam. Water. 2014; 6:3200–3223.

[7] Wang L, Zheng B, Zhang J, Liu X, Wu GY.Effects on euthrophication and hydrodynamics of Daning River after impoundment of Three Gorges Reservoir. Journal of Lake Sciences. 2012; 24:232–237.

[8] Rangel LM, Silva LHS, Rosa P, Roland F, Huszar VLM. Phytoplankton biomass is mainly controlled by hydrology and phosphorus concentrations in tropical hydroelectric reservoirs. Hydrobiologia. 2012; 693:13–28.

[9] Klug JL, Whitney K. Long-term trends in water quality in a New England hydroelectric impoundment. Northeastern Naturalist. 2015; 22:273–286.

[10] Withers PJA, Haygarth PM. Agriculture, phosphorus and eutrophication: a European perspective. Soil Use & Management. 2007; 23(supplement):1–4.

[11] Monzur Alam I, Abdallah S, Takashi A. Modelling multi-species algal bloom in a lake and inter-algal competitions. Water Science & Technology. 2009; 60:2599–2611. 19923766 10.2166/wst.2009.632

[12] Ghorbani M, Mirbagheri S, Hasani A, Nouri J, Monavari S. Algal bloom in aquatic ecosystems—an overview. Current World Environment. 2014; 9:105–108.

[13] Patiño R, Dan D, Vanlandeghem MM. Retrospective analysis of associations between water quality and toxic blooms of golden alga (*Prymnesium parvum*) in Texas reservoirs: Implications for understanding dispersal mechanisms and impacts of climate change. Harmful Algae. 2014; 33:1–11.

[14] Van dMD, Price KP. Harmful algal bloom characterization at ultra-high spatial and temporal resolution using small unmanned aircraft systems. Toxins. 2015; 7:1065–1078. 10.3390/toxins7041065 25826055 PMC4417955

[15] Trevor P, César FY, Frank KT. Marine ecology: Spring algal bloom and larval fish survival. Nature. 2003; 423:398–399. 10.1038/423398b 12761538

[16] Dorantes-Aranda JJ, Seger A, Mardones JI, Nichols PD, Hallegraeff GM.Progress in understanding algal bloom- mediated fish kills: the role of superoxide radicals, phycotoxins and fatty acids. Plos One. 2015;10(7):e0133549 10.1371/journal.pone.0133549 26197230 PMC4509671

[17] Hallett CS, Valesini FJ, Clarke KR, Hoeksema SD. Effects of a harmful algal bloom on the community ecology, movements and spatial distributions of fishes in a microtidal estuary. Hydrobiologia. 2015; 763:267–284.

[18] Tüzün I, Mason CF. Eutrophication and its control by biomanipulation: an enclosure experiment. Hydrobiologia.1996; 331:79–95.

[19] Domaizon I, Devaux J. A new approach in biomanipulation techniques: use of a phytoplanktivorous fish, the silver carp (*Hypophthalmichthys molitrix*). L Année Biologique. 1999; 38:91–106.

[20] Ekvall MK, Urrutiacordero P, Hansson LA. Linking cascading effects of fish predation and zooplankton grazing to reduced cyanobacterial biomass and toxin levels following biomanipulation. Plos One. 2014; 9(11):e112956–e112956. 10.1371/journal.pone.0112956 25409309 PMC4237340

[21] Guo JS, Chen Y, Li Z, Fang F, Sun ZY, Chen YB. Seasonal variation of chlorophyll a and its potential relationship with various algal species in Xiaojiang River backwater area, Three Gorges Reservoir. Environmental Science. 2011;32: 976–981. 21717735

[22] Waajen GWAM, Bruggen NCBV, Pires LMD, Lengkeekd W, Lürlinge M. Biomanipulation with quagga mussels (*Dreissena rostriformis bugensis*) to control harmful algal blooms in eutrophic urban ponds. Ecological Engineering. 2016; 90:141–150.

[23] Burns CW, Schallenberg M, Verburg P. Potential use of classical biomanipulation to improve water quality in New Zealand lakes: a re-evaluation. New Zealand Journal of Marine & Freshwater Research. 2014; 48:127–138.

[24] Radke RJ, Kahl U. Effects of a filter-feeding fish [silver carp, *Hypophthalmichthys molitrix* (Val.)] on phyto- and zooplankton in a mesotrophic reservoir: results from an enclosure experiment. Freshwater Biology. 2002; 47:2337–2344.

[25] Wang HJ, Liang XM, Jiang PH, Wang J, Shi-Kai W. TN: TP ratio and planktivorous fish do not affect nutrient-chlorophyll relationships in shallow lakes. Freshwater Biology. 2008; 53:935–944.

[26] Xu X, Tan Y, Yang G. Environmental impact assessments of the Three Gorges Project in China: Issues and interventions. Earth-Science Reviews. 2013; 124:115–125.

[27] MEPPRC(Ministry of Environmental Protection of the People’s Republic China). The environmental and ecological monitoring bulletins of the Three Gorges (2005–2015). http://jcs.mep.gov.cn/hjzl/sxgb/.

[28] Li B, Xu DD, Wang ZJ, Yue XJ, Zhang YG.Stable isotope (13C and15N) analysis of fish food web of the Xiaojiang Bay in Three Gorges Reservoir. Acta Ecologica Sinica. 2013; 33: 6704–6711.

[29] Hu L, Zou X, Zheng ZW, Zhang ZY, Pan XJ, Feng K, et al Study on coastal water quality in Xiaojiang River of Three Gorges Reservoir after experimental impoundment. Journal of Hydroecology. 2013; 32:1–18.

[30] Chen XJ, Pan XJ, Zou X, Hu L, Zhang ZY, Feng K et al Studies on the temporal and spatial variation of water environment in Xiaojiang River Backwater region of Three Gorges Reservoir. Journal of Hydroecology. 2012; 33:1–6.

[31] Zhang ZY, Wan CY, Zheng ZW, Hu L, Feng K, Chang JB, et al Plant community characteristics and their responses to environmental factors in the water level fluctuation zone of the three gorges reservoir in China. Environmental Science & Pollution Research. 2013; 20:7080–7091. 23589274 10.1007/s11356-013-1702-1

[32] Zhu TS, Cao T, Ni L, He L, Yi CL, Yuan CB, et al Improvement of water quality by sediment capping and re-vegetation with Vallisneria natans L.: A short-term investigation using an in situ enclosure experiment in Lake Erhai, China. Ecological Engineering. 2016; 86:113–119.

[33] Xie P. Silver carp and bighead carp, and their use in the control of algal blooms. Beijing: Science Press; 2003.

[34] Gibson JAE, Swadlings KM, Burton HR. Interannual variation in dominant phytoplankton species and biomass near Davis station, east Antarctica. Proceedings of the Nipr Symposium on Polar Biology. 1997; 10:77–89.

[35] Zhao HJ, Wang Y, Yang LL, Yuan LW, Peng DC. Relationship between phytoplankton and environmental factors in landscape water supplemented with reclaimed water. Ecological Indicators. 2015; 58, 113–121.

[36] Hu HJ, Li YY, Wei YX, Zhu HZ, Chen JY, Shi ZX. Freshwater Algae in China. Shanghai: Science Technology Press; 1980.

[37] Norušis MJ. SPSS 16.0 Guide to Data Analysis. 2nd ed USA: Prentice Hall Press; 2008.

[38] Hedges LV, Gurevitch J, Curtis PS. The meta-analysis of response ratios in experimental ecology. Ecology. 1999; 80: 1150–1156.

[39] Yi C, Guo L, Ni L, Luo C. Silver carp exhibited an enhanced ability of biomanipulation to control cyanobacteria bloom compared to bighead carp in hypereutrophic Lake Taihu mesocosms. Ecological Engineering. 2016; 89:7–13.

[40] Lieberman DM.Use of silver carp (*Hypophthalmichthys molotrix*) and bighead carp (*Aristichthys nobilis*) for algae control in a small pond: changes in water quality. Journal of Freshwater Ecology. 1996; 11:391–397.

[41] Zhang X, Xie P, Huang X. A review of nontraditional biomanipulation. Scientific World Journal. 2008; 8:1184–1196. 10.1100/tsw.2008.144 19082415 PMC5848641

[42] Ha JY, Saneyoshi M, Park HD, Toda H, Kitano S, Homma T et al Lake restoration by biomanipulation using piscivore and Daphnia stocking; results of the biomanipulation in Japan. Limnology. 2013; 14:19–30.

[43] Skov C, Perrow MR, Berg S, Skovgaard H. Seven years of stocking with piscivorous fish in a Danish shallow lake: changes in fish community and water quality. Freshwater Biology. 2002; 47:2388–2400.

[44] Ding RH. The fishes of Sichuan. China: Sichuan Publishing House of Science and Technology; 1994.

[45] Zanden MJV, Vadeboncoeur Y. Fishes as integrators of benthic and pelagic food webs in lakes. Ecology. 2008; 83:2152–2161.

[46] Sieben K, Ljunggren L. A meso-predator release of stickleback promotes recruitment of macroalgae in the Baltic Sea. Journal of Experimental Marine Biology & Ecology. 2011; 397:79–84.

[47] Ma H, Cui FY, Liu ZQ, Fan ZQ, He WJ, Yin PJ. Effect of filter-feeding fish silver carp on phytoplankton species and size distribution in surface water: A field study in water works. Journal of Environmental Sciences. 2010; 22:161–167. 10.1016/s1001-0742(09)60088-7 20397401

[48] Cremer MC, Smitherman RO. Food habits and growth of silver and bighead carp in cages and ponds. Aquaculture. 1980;20:57–64.

[49] Zhang X, Xie P, Hao L, Guo NC, Gong YG, Hu XL, et al Effects of the phytoplanktivorous silver carp (*Hypophthalmichthys molitrixon*) on plankton and the hepatotoxic microcystins in an enclosure experiment in a eutrophic lake, Lake Shichahai in Beijing. Aquaculture.2006; 257:173–186.

[50] Fukushima M, Takamura N, Sun L, Nakagawa M, Matsushige K, Xie P. Changes in the plankton community following introduction of filter-feeding planktivorous fish. Freshwater Biology. 2001; 42:719–735.

[51] Văsek M, Prchalová M, Peterka J, Ketelaars HAM, Wagenvoort AJ, Čech M, et al The utility of predatory fish in biomanipulationof deep reservoirs. Ecological Engineering. 2013; 52: 104–111.

[52] Vriesman ACVB, Kole BJ, Puylaert JBCM. The influence of biomanipulations (fish removal) on the structure of lake foodwebs, case studies using the LakeWeb-model. European Radiology. 2002; 37:87–99.

